# A continuum hypothesis of psychotomimetic rapid antidepressants

**DOI:** 10.1177/23982128211007772

**Published:** 2021-05-03

**Authors:** Joost Haarsma, Catherine J Harmer, Sandra Tamm

**Affiliations:** 1 Wellcome Centre for Human Neuroimaging, University College London, London, UK; 2 Department of Psychiatry and Oxford Health NHS Foundation Trust, Warneford Hospital, University of Oxford, Oxford, UK; 3 Stress Research Institute, Department of Psychology, Stockholm University, Stockholm, Sweden; 4 Department of Clinical Neuroscience, Karolinska Institute, Stockholm, Sweden

**Keywords:** Ketamine, sleep deprivation, psychedelics, rapid antidepressants, predictive coding, psychotomimetics

## Abstract

Ketamine, classical psychedelics and sleep deprivation are associated with rapid effects on depression. Interestingly, these interventions also have common psychotomimetic actions, mirroring aspects of psychosis such as an altered sense of self, perceptual distortions and distorted thinking. This raises the question whether these interventions might be acute antidepressants through the same mechanisms that underlie some of their psychotomimetic effects. That is, perhaps some symptoms of depression can be understood as occupying the opposite end of a spectrum where elements of psychosis can be found on the other side. This review aims at reviewing the evidence underlying a proposed *continuum hypothesis of psychotomimetic rapid antidepressants*, suggesting that a range of psychotomimetic interventions are also acute antidepressants as well as trying to explain these common features in a hierarchical predictive coding framework, where we hypothesise that these interventions share a common mechanism by increasing the flexibility of prior expectations. Neurobiological mechanisms at play and the role of different neuromodulatory systems affected by these interventions and their role in controlling the precision of prior expectations and new sensory evidence will be reviewed. The proposed hypothesis will also be discussed in relation to other existing theories of antidepressants. We also suggest a number of novel experiments to test the hypothesis and highlight research areas that could provide further insights, in the hope to better understand the acute antidepressant properties of these interventions.

## Introduction

Depression is a world-wide leading cause of disability (World Health Organization, 2017). For both standard antidepressants, such as selective serotonin reuptake inhibitors (SSRIs), and psychological treatments, a significant treatment effect is generally only seen after weeks of treatment. Contrary, a number of less well-established treatments, including ketamine, psychedelics and sleep deprivation are shown to be associated with a rapid decrease in depressive symptoms (Bobo et al., 2016; Muttoni et al., 2019; Riemann et al., 2020).

One of the most popular theories for the neurocognitive effects of traditional antidepressants states that they target the affective biases commonly seen in depression (Beck et al., 1979). This negativity bias leads to a perception of the surrounding world in pessimistic terms, which eventually leads to a depressive worldview. By altering the affective bias, the depressive worldview becomes corrected over time (Harmer et al., 2009).

Cognitive inflexibility is a separate component of depression, believed to be an important treatment target (Rock et al., 2014; Solé et al., 2015). Cognitive flexibility is commonly defined as the ability to switch between different types of tasks or concepts (Diamond, 2013), but we here also refer to cognitive inflexibility as a broader inability to adjust one’s thinking from old situations to new situations. This aspect of depression likely interacts with the affective bias account described above, because in order to change a depressive worldview, a certain amount of flexibility in this worldview is required, such that expectations about negative and positive events can be corrected. Speculatively, this could indicate that traditional antidepressants potentially only affect cognitive inflexibility indirectly, which might contribute to the reason why they take multiple weeks to have their effect. This would be in line with the resource-allocation hypothesis by Park et al. (2018), which suggests that the ability of early changes in ‘cold’ cognition (e.g. executive functioning) to predict treatment response in depression is dependent on its association with ‘hot’ cognition, that is, cognitive-emotional functions.

Interestingly, ketamine, psychedelics and sleep deprivation have in common that they are acute antidepressants, as well as that they induce some of the symptoms of psychosis. These include in the case of ketamine distorted sense of self and thought, as well as experiences of dissociation (Krystal et al., 1994), whereas the effects of psychedelics are more perceptual but can also include experiences of distorted thinking (Gouzoulis-Mayfrank et al., 1998; Vollenweider and Kometer, 2010). Sleep deprivation causes progressively more severe symptoms of psychosis, starting with perceptual alterations initially, and distortions in thinking if sleep deprivation is maintained (Waters et al., 2018). However, there are also important differences between psychotic symptoms caused by ketamine, psychedelics and sleep deprivation, compared to clinical cases of psychosis, where the latter tends to involve more auditory hallucinations (Bauer et al., 2011), which are rare under the influence of ketamine and psychedelics. In contrast, psychedelics tend to affect the visual modalities more strongly, although distortions in self-awareness and thinking are present under higher doses as well (Leptourgos et al., 2020). Furthermore, clinical psychosis is often characterised by a lack of insight which is usually preserved under the influence of drugs (Leptourgos et al., 2020; Pomarol-Clotet et al., 2006). Interestingly, psychosis (in particular the early phases) has been commonly described to reflect an overly flexible brain (Fletcher and Frith, 2009; Sterzer et al., 2018). If cognitive inflexibility is indeed an important feature of depression, and psychotomimetic interventions increase cognitive flexibility, this raises the exciting possibility that psychotomimetics like ketamine, psychedelics and sleep deprivation have acute antidepressant effects precisely because of their ability to increase cognitive flexibility.

In the following review, we will formulate the hypothesis that there is a shared mechanism that underlies the psychotomimetic properties and acute antidepressant effects of these treatments. First, we will describe the various acute antidepressants and their psychotomimetic features. We will subsequently review these treatments through the theoretical framework referred to as predictive coding theory (Friston, 2010; Spratling, 2017) which we suggest can help us understand why psychotomimetic interventions might be acute antidepressants. Finally, we will compare our suggested hypothesis with existing theories and make suggestions for future studies to test the validity of the hypothesis.

## An overview of acute antidepressants with psychotomimetic effects

### Ketamine

Ketamine is a non-competitive open channel N-methyl-D-aspartate (NMDA) antagonist, first synthesised in 1962 by Calvin Stevens at Parke-Davis Co and as an anaesthetic used in humans since 1965 (Gao et al., 2016; Mion, 2017). During the last two decades, sub-anaesthetic dosage of ketamine has repeatedly been shown to cause an acute antidepressant effect (Berman et al., 2000; Bobo et al., 2016; Kishimoto et al., 2016). The effect is seen within hours and peaking at 24 h after administration, with a decline during the following week (Bobo et al., 2016). A number of meta-analyses have investigated the efficacy in treatment showing overall large effect sizes for the acute effect (McGirr et al., 2015; Newport et al., 2015; Xu et al., 2016). Although the treatment is associated with some side-effects, including psychotomimetic symptoms, these generally resolve within hours after administration (Bobo et al., 2016).

Ketamine administration causes a burst in glutamate via inhibition of gamma-aminobutyric acid (GABA) interneurons, followed by a cascade involving stimulated AMPA receptors and activation of calcium channels leading to a release of brain-derived neurotrophic factor (BDNF) and mammalian target of rapamycin complex–1 (mTORC1) signalling, and in turn an increased synthesis of synapse proteins (Autry et al., 2011; Li et al., 2010; Moghaddam et al., 1997). The exact pathway that mediates the effect of ketamine on depressive symptoms is however not clear, even though an increase in synaptic plasticity has been suggested as one possible mechanism (Krystal et al., 2013; Monteggia et al., 2013).

As indicated above, ketamine is also associated with psychotomimetic effects, which has been shown both as side-effects in relation to anaesthesia (Bobo et al., 2016) and in experimental studies in healthy volunteers (Krystal et al., 1994; Malhotra et al., 1996). The latter has made it a useful model for psychosis (Corlett et al., 2007). Psychotomimetic effects are often manifested as alterations in perception, dissociations and hallucinations (Corlett et al., 2007, 2016; Krystal et al., 1994), but some studies have also emphasised the ability of ketamine to mimic the negative symptoms of schizophrenia including apathy and social withdrawal (Anis et al., 1983; Krystal et al., 1994).

### Psychedelics

Classical psychedelics, that is, psychedelics that exert their effects through 5HT2a receptor agonism, have a long history of usage in spiritual practices, ranging as far back as 5700 years ago in native Mexican cultures (Bruhn et al., 2002). Psychedelics became popular in the West in the 1950s and 1960s, which was accompanied by a surge in research into the properties of the drug (Reiff et al., 2020). In the last decade, there has been an increasing interest in the use of classic psychedelics in treating individuals with treatment-resistant depression (Reiff et al., 2020). Psilocybin, ayahuasca and lysergic acid diethylamide (LSD) have been shown to reduce depressive symptoms and anxiety as single treatments, but more often in combination with psychotherapy (Carhart-Harris et al., 2016, 2018a; de Osório et al., 2015; Reiff et al., 2020).

Psychedelics exert their effects by increasing glutamatergic activity following activation of pyramidal neurons, primarily in layer 5 of the cortex (Aghajanian and Marek, 1996, 1999; Béïque et al., 2007). Initially this was believed to be due to activation of the presynaptic 5HT2a receptors (González-Maeso et al., 2007; Puig et al., 2003), but more recent studies have pointed to postsynaptic receptors (González-Maeso et al., 2007; Puig et al., 2003). This activation can subsequently lead to downstream effects on serotonergic and dopaminergic activity in the raphe nucleus and ventral tegmental area following activation of 5HT2a receptors in the medial prefrontal cortex (Vollenweider and Kometer, 2010). Indeed, following the intake of psilocybin, increases in dopamine levels in the striatum, which correlated to euphoria and depersonalisation, have been shown (Vollenweider et al., 1999). Speculatively, psychedelics have furthermore been suggested to cause neuroplastic adaptations that might underlie some of the therapeutic effects (Vollenweider and Kometer, 2010).

Psychedelics induce pronounced changes in consciousness, including changes in perceptual experiences such as brightening of colours, synaesthesia, illusory patterns, or full-blown hallucinations and a sense of derealisation (Hoch et al., 1952; Leptourgos et al., 2020; Leuner and Holfeld, 1962). These experiences are not unlike the experiences sometimes described in the early stages of clinical psychosis, sometimes referred to as the prodromal period, where patients often experience a heightening of the senses, and an inability to dissociate the important from the unimportant (Bowers and Freedman, 1966; Chapman, 1966; Gouzoulis-Mayfrank et al., 1998).

### Sleep deprivation

Sleep deprivation as an antidepressant treatment was first reported in 1818, by the German psychiatrist Johann Christian August Heinroth (Steinberg and Hegerl, 2014). In the mid-1960s, the antidepressant effects of sleep loss was suggested in a case study of a teacher whose depression eased after sleepless nights (Schulte, 1966) and the first trial of sleep deprivation for depression was published in 1971 (Pflug and Tölle, 1971). A meta-analysis published in 2017 combining 66 studies showed an overall response rate to sleep deprivation in depression of 45%–50% of patients (Boland et al., 2017). Notably, most studies of sleep deprivation as a treatment for depression have been performed with little experimental control, such as randomisation or blinded assessments. In most patients, the depressive symptoms, also, revert after sleep.

A number of putative mechanisms have been suggested to cause the antidepressant effect of sleep deprivation. Bunney and Bunney (2013) proposed that the effect is mediated via CLOCK gene transcription and a ‘reset’ of the circadian rhythm. Wolf and colleagues formulated a synaptic plasticity model theory which suggests that sleep deprivation enhances cortical synaptic strength and facilitates long-term potentiation, through homeostatically shifting the brain to a window of long-term potentiation inducibility (Wolf et al., 2015). A handful of studies investigated changes in neurotransmitters in association to therapeutic sleep deprivation, suggesting a possible role for serotonin (Lopez-Rodriguez et al., 2003; Salomon et al., 1994), dopamine (Benedetti et al., 1996, 2001; Ebert et al., 1994) or the glutamatergic system (Murck et al., 2009).

Analogous with ketamine and psychedelics, sleep deprivation is associated with psychotic symptoms, in healthy volunteers (Waters et al., 2018) but even more pronounced in patients with bipolar disorder (Lewis et al., 2017; Wehr et al., 1987). A recent review suggested that psychotic symptoms after sleep deprivation develop in an almost dose-dependent manner with perceptual distortions, anxiety, irritability, depersonalisation and temporal disorientation starting within 24–48 h of sleep loss, followed by complex hallucinations and disordered thinking after 48–90 h, and delusions after 72 h (Waters et al., 2018).

## A theoretical framework to understand the link between psychosis and rapid antidepressants

The previous section described interventions that are both rapid antidepressants as well as psychotomimetic. In order to understand what kind of mechanisms underlie this phenomenon, we need to have a framework for how the brain forms a model of the world in the first place, such that we can start to see how changes in that model can lead to both depression-like symptoms and psychosis. The model we will use in this article is the hierarchical predictive coding model (Friston, 2009, 2010; Friston and Kiebel, 2009; Rao and Ballard, 1999; Spratling, 2008, 2010, 2017).

Predictive coding is a solution to how the brain forms a model of its environment on an algorithmic and neural level (Clark, 2015; Hohwy, 2013). Although various iterations of the hierarchical predictive coding model exist, they have in common the idea that the brain forms a hierarchical generative model of its environment where predictions are generated about the most likely causes of sensory information. These predictions are compared to new sensory input. When there is a mismatch between the two, a prediction error is generated that updates the brain’s predictions about the sensory input, which over time improves the brain’s model of its environment. Importantly, both predictions and sensory input are represented by probability distributions which have a mean and variance. The mean can be seen as the brain’s estimate of the belief whereas the variance is an estimate of how certain this belief is. This certainty is often referred to as the precision of sensory input (in the case of new evidence) or priors (in the case of predictions) and is used to weight the influence of higher-level beliefs versus lower-level evidence (Friston, 2009; Friston and Kiebel, 2009; Rao and Ballard, 1999). For example, a self-image might be stable (precise prior), and unchangeable in the light of evidence, or flexible and responsive to change (imprecise/flexible prior). Experimentally, it can be difficult to disambiguate whether a behavioural phenomenon is due to increased precision of sensory input or a flexibility of priors, as they tend to result in similar experiences (Haarsma et al., 2020c).

Certain neurotransmitters are believed to play an important role in coding of the precision of sensory evidence, as well as the precision of prior expectations. For example, dopamine has been associated with coding the precision of prediction errors (Diederen et al., 2017; Haarsma et al., 2020a) as well as acetylcholine, which has been associated with increasing reliance on bottom-up information (Baldeweg et al., 2006; Moran et al., 2013). In contrast, the NMDA-receptors that ketamine binds to have been suggested to play a role in coding the precision of prior beliefs (Sterzer et al., 2018).

Relevant to the current theoretical perspective is that there are various ways in which learning about the world can go awry. First, there can be changes in the degree to which new information is given weight. For example, when negative information is given more weight when learning about our environment, a more negative worldview will emerge. This is what is known as an affective bias, which is thought to contribute to depression, and is suggested to be altered by SSRIs (Harmer et al., 2009). In contrast, the prior expectation can be given undue weight, such that it is inflexible to new changes. For example, in depression, one might expect negative events to be very likely to occur and positive events likely to be absent. When these expectations are overly precise, this could lead to a phenomenon of cognitive inflexibility, which is known to contribute to depression (Coifman and Summers, 2019; Leahy et al., 2012).

## Psychosis and depression as disorders of precision

### Psychosis

If the brain indeed forms a model of the world through a process of hierarchical inference, where precision of prior expectations, sensory input and prediction errors is key, erroneous models of the world can be the result of misallocation of precision, resulting in symptoms of hallucinations and delusions as commonly seen in psychosis. One of the earliest accounts suggested that strong prior expectations might be the cause of hallucinations and delusions (Stephan et al., 2006), whereas others suggested that psychosis can be understood as a case of weaker prior expectations resulting in overly precise prediction errors (Fletcher and Frith, 2009). More recent accounts aimed to reconcile these different accounts by suggesting that they might be differently related to state and trait symptoms (Adams et al., 2013) and the most recent reiteration of the hierarchical predictive coding model of psychosis suggested that psychosis can best be understood as a result of aberrant changes in precision in different levels of the cortical hierarchy through different stages of psychotic illness (Sterzer et al., 2018). Specifically, precision of prior expectations is suggested to be weakened in early perceptual processes perhaps more pronounced in the early stages of illness, whereas the precision of higher order learned beliefs becomes stronger and can start to dominate perception (Sterzer et al., 2018). Indeed, there are various lines of evidence supporting this view, demonstrating weakened perceptual priors in psychosis (Dima et al., 2010; Haarsma et al., 2020b; Schmack et al., 2013), as well as stronger learned priors (Cassidy et al., 2018; Haarsma et al., 2020b; Teufel et al., 2015; Valton et al., 2019).

### Depression

There are multiple variations of the hierarchical predictive coding model of depression, which emphasise different features of depression and go into varying levels of detail. However, there is a high degree of overlap between the models, in the sense that they all involve an increased influence of prior expectations on cognitive as well as interoceptive processes. It has been argued that predictive coding models are particularly applicable to depression, as the core symptoms seem to be centred on expecting negative events to occur with high likelihood as well as positive events with low likelihood (Kube and Rozenkrantz, 2020). Understanding how these prior expectations can be altered therefore seems to be a crucial component of understanding the core mechanisms underlying depression and developing novel treatments (Kube and Rozenkrantz, 2020). Others have emphasised the importance of estimating uncertainty in predictive coding models, and that an inability to reduce uncertainty seems to be particularly related to anxiety and depressive symptoms, whereas reducing uncertainty is related to positive emotions (Clark et al., 2018). Another view puts the emphasis on aberrant interceptive predictions (Barrett et al., 2007). Here it is argued that there is a deficit in minimising prediction errors in the limbic system as a result of prolonged stress, which in time generates false interoceptive predictions about autonomic, metabolic and immunological requirements. In other words, these predictions generate false stress responses, effectively dysregulating the hypothalamic–pituitary–adrenal (HPA)-axis. Sickness behaviour and negative affect are subsequently used by the body to reduce energy expenditure, which overtime results in depression. Thus, depression is viewed here as a perturbation in energy regulation and interoceptive inference, that is, the prediction errors and associated precision (Barrett et al., 2016).

Other models focus on reward learning. Here depressed brains are characterised by a hierarchical structure of depressive beliefs, producing a consistent negative bias. This bias leads to oversampling the environment to confirm these negative expectations (Chekroud, 2015). In similar vein, depression has been argued to reflect impaired reward-approach behaviours, as a result of having prior beliefs reflecting positive outcomes being unlikely (Joffily and Coricelli, 2013). These views echo earlier affective bias accounts (Harmer et al., 2009), although these earlier accounts emphasise changes in the sensory input rather than prior predictions. Recently, a predictive coding perspective has integrated cognitive immunisation, the tendency to reappraise evidence that disconfirms negative views about oneself, with overgeneralisation of negative feedback (Kube and Rozenkrantz, 2020), suggesting that these can be understood as overly strong negative prior expectations suppressing positively valenced prediction errors and exaggerated signalling of negatively valenced prediction errors (Kube and Rozenkrantz, 2020).

Taken together, these models suggest that symptoms of depression can be understood as overly precise prior expectations. These can be valence-specific where an overly strong, negatively valenced prior can cast everything in a negative light, whereas priors regarding positively valenced events can be such that these are not to be expected, thereby blunting reward responses. This maps onto the cognitive inflexibility that plays an important role in depression (Davis and Nolen-Hoeksema, 2000; Houston et al., 2004; Joormann et al., 2011; Remijnse et al., 2009, 2013). That is, an overly precise prior is less resistant to change, and making the priors more flexible might therefore be of therapeutic value (Badcock et al., 2017; Barrett et al., 2016; Edwards and Koob, 2012).

## Do psychotomimetics have antidepressant effects by decreasing precision of priors?

**Figure 1.**
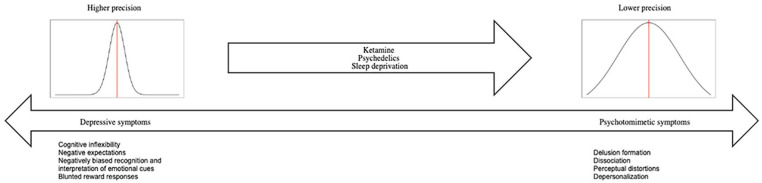
An overview of the continuum hypothesis of psychotomimetic rapid antidepressants.

So far, we have described that sleep deprivation, classical psychedelics and ketamine have a tendency to induce symptoms of psychosis in healthy individuals, as well as acute antidepressant effects in depressed patients. Other examples of interventions that are both (acute) antidepressant as well as known to induce some psychosis-like symptoms are meditation and isolation-tanks, but these are not further discussed in this article (Feinstein et al., 2018; Heuschkel and Kuypers, 2020; Kjellgren et al., 2008; Kuijpers et al., 2007). It could be argued that this link between the tendencies for these interventions to induce psychotic-like phenomenon might be coincidental to the antidepressant effect. However, an alternative explanation might be that the mechanisms for these substances and interventions to induce psychosis and have rapid antidepressant effects are the same, through making the brain’s prior expectations more flexible and malleable to change. In predictive coding terms, this means that these interventions might loosen the brain’s prior beliefs, which can induce a state of psychosis in healthy individuals, whereas it might push an excessively rigid brain into a more normal state of flexibility, reducing depressive symptoms. We will onwards present arguments for this view, which we will refer to as *the continuum hypothesis of psychotomimetic rapid antidepressants*, summarised in Figure 1.

### Ketamine

The evidence that supports the hypothesis that ketamine reduces precision of prior expectations mostly comes from psychosis research. In hierarchical predictive coding, the NMDA-receptor, on which ketamine acts, has been linked to coding the precision of prior expectations (Corlett et al., 2007, 2016). It is argued that ketamine alters inference by effectively making prior expectations more flexible and therefore more malleable by new experiences and that ketamine can induce symptoms of early psychosis through reducing the precision of prior expectations in otherwise healthy individuals. Neuroimaging studies have indeed shown aberrant prediction errors in the right inferior frontal cortex following ketamine infusion similar to what is seen in psychosis (Corlett et al., 2006). Furthermore, a sense of agency has been suggested to rely on suppression of prediction errors, which is likewise disturbed in both psychosis and after ketamine (Moore et al., 2011). Research on more higher-level learning and ketamine has demonstrated similar findings, in line with a reduced precision of prior expectations. For example, Vinckier et al. (2016) demonstrated that a single dose of ketamine changes the degree to which confidence modulates decision making, leading to a state of increased flexibility during learning. These effects on confidence were related to altered activity in the anterior cingulate cortex, which has previously been related to the therapeutic effects of ketamine on anhedonia (Lally et al., 2014, 2015; Morris et al., 2020). This suggests a potential link between cognitive flexibility and anhedonia that could be investigated in further research. Others (Weber et al., 2020) combined a roving mismatch paradigm with computational modelling, showing that prediction errors related to higher level statistics, that is, the volatility with which the probability of a mismatch could occur, were specifically perturbed. Coherently, using a similar computational model, other studies have found that there is an overestimation of volatility in schizophrenia (Deserno et al., 2020; Kaplan et al., 2016) and individuals at risk for psychosis (Cole et al., 2020).

Neurocognitive studies focusing on emotion processing after ketamine administration have shown reduced neural reactivity in the bilateral amygdalo-hippocampal complex during emotional stimulation (Scheidegger et al., 2016), a decrease in amygdala reactivity after ketamine for both positive and negative emotional face processing (Loureiro et al., 2020), and enhanced sensitivity for rewarded items accompanied by increased activity of reward-related brain regions in patients with depressive disorder (Sterpenich et al., 2019), findings that are not necessarily related to the brain becoming more flexible. Our *continuum hypothesis of psychotomimetic rapid antidepressants* suggests that ketamine’s rapid antidepressant effects are due to the substance’s ability to put the brain in a more flexible state. As mentioned above, cognitive inflexibility is an important part of depression, considered not to be a result of affective symptoms but representing a distinct sub-component of depression (Rock et al., 2014). Furthermore, cognitive deficits, including cognitive inflexibility, also predict treatment outcome and have been considered a treatment target (Rock et al., 2014; Solé et al., 2015). Interestingly, although ketamine has been found to impair cognitive functioning in healthy individuals on a wide range of tasks (Adler et al., 1998; Krystal et al., 2005; Malhotra et al., 1996), when given to individuals with depression, there are pro-cognitive effects found on simple processing speed tasks, as well as cognitive flexibility (Basso et al., 2020; Zheng et al., 2019). Other studies have also found differing effects of ketamine on depressive symptoms in healthy and patients with depression, showing increases in depression in healthy individuals following ketamine, whereas lowered depression was found in patients (Nugent et al., 2019). In summary, we suggest that ketamine reduces the precision of prior expectation during inference, and thereby reduces the cognitive biases, for example, cognitive inflexibility and overly precise negative and positive expectations, in depression.

### Psychedelics

In similar fashion, psychedelics have long been suggested to make the mind more flexible. The most recent iteration of this model has been referred to as the REBUS model, which stands for relaxed beliefs under psychedelics (Carhart-Harris and Friston, 2019). The evidence in favour of this model primarily comes from experiments related to sensory preprocessing, that are reinterpreted in a predictive coding framework. For example, LSD reduces mismatch negativity responses (Timmermann et al., 2018), interpreted as reduced precision in prior expectations. Similarly, the Kanizsa illusion, which is believed to rely on top-down feedback from higher visual regions (Kok et al., 2016; Pak et al., 2020), is reduced under psilocybin (Kometer et al., 2011) and binocular rivalry is altered during the psychedelic state (Carter et al., 2005, 2007). Furthermore, pre-pulse inhibition is reduced under psychedelics (Quednow et al., 2012; Schmid et al., 2015) and increased bottom-up signalling from the parahippocampus to the visual cortex during music listening was observed after LSD (Kaelen et al., 2016).

As with ketamine, some studies explored emotional processing in relation to psychedelics. For example, Müller et al. found reduced reactivity of the left amygdala and the right medial prefrontal cortex relative to placebo during the presentation of fearful faces after LSD (Mueller et al., 2017), whereas Roseman et al. (2018) showed the opposite pattern with increased amygdala responses to both negative and positive stimuli after psilocybin. Whereas the former finding supports an effect more in line with a change in affective biases, the latter finding might be more easily explained as a valence nonspecific upregulation of the brain’s responses to both positive and negative stimuli, which could reflect a more flexible brain state. This is in line with what has recently been found in a behavioural study where the learning rates for both rewards and punishments were increased after taking LSD, which again is in line with a reduction in the precision of the brain’s prior expectations (Kanen et al., 2021). Future studies can use computational models to explore whether this is indeed explained by reductions in prior precision (see section ‘How does the continuum hypothesis fit with existing theoretical perspectives?’).

In summary, there is some indirect evidence that psychedelics reduce the precision of prior expectations in sensory processing, although studies focusing on cognition have been limited due to the difficulties of doing behavioural experiments with psychedelics, that is, participants becoming disengaged (Carhart-Harris et al., 2018b).

### Sleep deprivation

Various studies have looked into the effects of sleep deprivation on cognition and emotion, usually conducted in healthy individuals focusing on impairments in attention, emotion processing and decision making (Killgore, 2010; Krause et al., 2017; Lowe et al., 2017). In healthy volunteers, the most prominent effects of sleep deprivation are an increase in sleepiness as well as decreased performance in attentional tasks in a dose-dependent manner (Lowe et al., 2017). Furthermore, sleep deprived healthy subjects make more risky decisions, assign greater weights to more recent rewards (Killgore et al., 2006; Olson et al., 2016), and show lower response inhibition and cognitive control (Acheson et al., 2007; Cedernaes et al., 2014; Demos et al., 2016). Subcortical reward‑related regions of the brain also seem to become hypersensitised by acute sleep deprivation (Gujar et al., 2011; Krause et al., 2017). However, evidence directly supporting the notion that prior expectations become more flexible following sleep deprivation is lacking. Hence, there is some evidence suggesting that sleep deprivation causes an increase in the AMPA–NMDA ratio (McDermott et al., 2006). It has been argued that prior expectations are realised top-down via NMDA and GABA signalling and mismatch between those priors and incoming information is signalled bottom-up via AMPA receptors (Corlett et al., 2016), which would in turn indicate that sleep deprivation would lead to a favouring of bottom-up signalling. It is however unclear whether these alterations might make depressed people more cognitively flexible or underlie the psychotomimetic effects of sleep deprivation.

## How does the continuum hypothesis fit with existing theoretical perspectives?

In the previous section, we presented *the continuum hypothesis of psychotomimetic rapid antidepressants* and reviewed some evidence underlying the hypothesis. In this section, we will further discuss how this hypothesis can be tested on a clinical, behavioural and neural level.

First, our hypothesis predicts that the precision of prior expectations, of both negative and positive events, is reduced by the interventions described above. Although there is some indirect evidence supporting this, few studies have addressed how these interventions alter the precision of prior expectations regarding negative and positive events, and to what extent this underlies the acute antidepressant effects. Future studies could consequently use behavioural paradigms that allow the study of how precision of prior expectations change during inference, in healthy volunteers and more importantly in patients with depression. Reversal learning paradigms where expectations about negative and positive events are learned over time might be particularly useful for this purpose (Browning et al., 2015; Pulcu and Browning, 2017). For example, see Pulcu and Browning (2017) for a paradigm where an agent was required to respond adaptively to fluctuations in the volatility of both losses and rewards. It has been argued before that affective disorders might manifest themselves through altered signalling of different forms of uncertainty (Pulcu and Browning, 2019). We predict that ketamine, psychedelics and sleep deprivation could reduce the precision of prior expectations in such tasks, making the patient more receptive to upcoming rewards and losses and expect this to be related to the antidepressant effects. This again highlights the interesting parallel to clinical effects of ketamine on anhedonia and reward processing. Computational models that allow for the modelling of higher order prior expectations during inference could be especially suitable for these experiments (Iglesias et al., 2013; Mathys et al., 2011, 2014; Piray and Daw, 2020).

Second, we expect that effects of ketamine, psychedelics and sleep deprivation are separable from the effects of commonly used SSRIs, in that they to a larger extent directly alter the use of information that has already been acquired (i.e. priors and their precision) (Stuart et al., 2015). This would be in line with what others have suggested to be the effects of ketamine (Stuart et al., 2015), but in contrast with a hypotheses that suggest that ketamine works on the same mechanism as SSRIs but acts faster (Hales et al., 2017).

The model presented in this article is suggested to provide a framework for how to understand how psychotomimetic rapid antidepressants can be further understood. There are however a number of potential caveats to our suggested model, and the model does not provide an explanation for how the different treatments should be understood at a molecular/neurochemical level. Whether the suggested model can be linked to work suggesting expression of genes/growth factors (Bunney and Bunney, 2013), functional connectivity (Bosch et al., 2013; Carhart-Harris et al., 2017; Scheidegger et al., 2012; Timmermann et al., 2018) and/or alteration in the immune system (Irwin et al., 2016; Loix et al., 2011) as mechanisms for the different treatment is also not known, and in fact these underlying mechanisms might differ between treatments.

Furthermore, even in terms of neurocognitive research, the *continuum hypothesis of psychotomimetic rapid antidepressants* needs to be tested against other possible theories. For example, we have suggested that the interventions described above alter the way negatively and positively valenced prior expectations shape inference. However, there are various other ways they could influence prior expectations, which are not limited to negatively or positively valenced information per se. Rather than influencing the expectations of valenced information, ketamine, as a well-known dissociative, might affect interoceptive signalling, which has been hypothesised to play an important role in depression (Barrett et al., 2016). Furthermore, rather than suggesting that these interventions decrease the effect of negative memories or experiences, our model suggests that the influence of both negative and positive expectations will be diminished by the interventions discussed, putting the brain in a more flexible state, allowing change to occur. In these more flexible brain states, we might paradoxically find that brain and behavioural responses to positive as well as negative outcomes can be upregulated (Kanen et al., 2021; Roseman et al., 2018). This might highlight the importance of combining these substances with, for example, psychological interventions.

Finally, we have made the case that psychedelics, ketamine and sleep deprivation are psychotomimetic, as well as acute antidepressants, and that this might relate to the ability to lower the precision of prior expectations. However, this is not to say that it is the psychotomimetic effects that are the key driving factor underlying the antidepressant effects. Instead, psychosis might be an excessive state of heightened cognitive flexibility. Indeed, some studies using ketamine have found no relationship between the psychotic-like effects ketamine induces and the therapeutic effects of the drug (Acevedo-Diaz et al., 2020), whereas others found the opposite pattern (Sos et al., 2013). Thus, it would be as possible that the psychosis and acute antidepressant model share that their explanation relies on flexibility of prior expectations, but does not necessarily entail that the state of psychosis is itself antidepressant. It should also be highlighted that depressive and psychotomimetic symptoms do not necessarily translate to specific diagnoses.

Keeping all the above-mentioned limitations in mind, the proposed hypothesis might still be informative when developing future treatments. For example, psychotomimetic side-effects of new treatments might in some cases be seen acceptable, at least at an early stage of development. Furthermore, incorporating the *continuum hypothesis of psychotomimetic rapid antidepressants* with existing theories, including affective bias, might provide a better understanding of how different treatments might interact. As, for example, the combination of fast-acting antidepressants with psychotherapy might lead to additive effects, since there may be greater adaptation to new information gained through therapy. Indeed, psychedelics, and to some extent ketamine, are often used in combination with psychotherapy, supporting this view (Carhart-Harris et al., 2016, 2018a; de Osório et al., 2015; Dore et al., 2019; Reiff et al., 2020).

## Conclusion

In summary, we have reviewed various rapid antidepressants, primarily ketamine, classical psychedelics and sleep deprivation, that also have psychotomimetic properties. We furthermore suggest that this link between the tendency for rapid antidepressants to be psychotomimetic is not necessarily accidental, but instead might rely on their propensity to diminish the brain’s priors across different levels of the cortical hierarchy and we refer to this idea as *the continuum hypothesis of psychotomimetic rapid antidepressants*. In healthy individuals, this results in more weight being put on new information, that is, exaggerated bottom-up prediction errors, effectively causing an overly flexible state of mind and leading to psychotic symptoms. In patients with depression, increasing the flexibility of the brain’s prior expectations might, on the contrary, remediate the cognitive inflexibility characterising the disorder, in almost an immediate fashion.

## References

[bibr1-23982128211007772] Acevedo-Diaz EE Cavanaugh GW Greenstein D, et al. (2020) Can ‘floating’ predict treatment response to ketamine? Data from three randomized trials of individuals with treatment-resistant depression. Journal of Psychiatric Research 130: 280–285. DOI: 10.1016/j.jpsychires.2020.06.012. 32861983 10.1016/j.jpsychires.2020.06.012 PMC8073211

[bibr2-23982128211007772] Acheson A Richards JB de Wit H (2007) Effects of sleep deprivation on impulsive behaviors in men and women. Physiology and Behavior 91(5): 579–587. 17477941 10.1016/j.physbeh.2007.03.020

[bibr3-23982128211007772] Adams RA Stephan KE Brown HR, et al. (2013) The computational anatomy of psychosis. Frontiers in Psychiatry 4: 47. DOI: 10.3389/fpsyt.2013.00047. 23750138 10.3389/fpsyt.2013.00047 PMC3667557

[bibr4-23982128211007772] Adler CM Goldberg TE Malhotra AK, et al. (1998) Effects of ketamine on thought disorder, working memory, and semantic memory in healthy volunteers. Biological Psychiatry 43(11): 811–816. 9611670 10.1016/s0006-3223(97)00556-8

[bibr5-23982128211007772] Aghajanian GK Marek GJ (1996) Serotonin induces excitatory postsynaptic potentials in apical dendrites of neocortical pyramidal cells. Neuropharmacology 36(4–5): 589–599. 10.1016/s0028-3908(97)00051-8 9225284

[bibr6-23982128211007772] Aghajanian GK Marek GJ (1999) Serotonin, via 5-HT(2A) receptors, increases EPSCs in layer V pyramidal cells of prefrontal cortex by an asynchronous mode of glutamate release. Brain Research 825(1–2): 161–171. 10216183 10.1016/s0006-8993(99)01224-x

[bibr7-23982128211007772] Anis NA Berry SC Burton NR, et al. (1983) The dissociative anaesthetics, ketamine and phencyclidine, selectively reduce excitation of central mammalian neurones by N-methyl-aspartate. British Journal of Pharmacology 79(2): 565–575. 6317114 10.1111/j.1476-5381.1983.tb11031.x PMC2044888

[bibr8-23982128211007772] Autry AE Adachi M Nosyreva E, et al. (2011) NMDA receptor blockade at rest triggers rapid behavioural antidepressant responses. Nature 475(7354): 91–96. 21677641 10.1038/nature10130 PMC3172695

[bibr9-23982128211007772] Badcock PB Davey CG Whittle S, et al. (2017) The depressed brain: An evolutionary systems theory. Trends in Cognitive Sciences 21(3): 182–194. 28161288 10.1016/j.tics.2017.01.005

[bibr10-23982128211007772] Baldeweg T Wong D Stephan KE (2006) Nicotinic modulation of human auditory sensory memory: Evidence from mismatch negativity potentials. International Journal of Psychophysiology 1: 49–58. DOI: 10.1016/j.ijpsycho.2005.07.014. 10.1016/j.ijpsycho.2005.07.014 16313986

[bibr11-23982128211007772] Barrett LF Mesquita B Ochsner KN, et al. (2007) The experience of emotion. Annual Review of Psychology 58(1): 373–403. 10.1146/annurev.psych.58.110405.085709 PMC1934613 17002554

[bibr12-23982128211007772] Barrett LF Quigley KS Hamilton P (2016) An active inference theory of allostasis and interoception in depression. Philosophical Transactions of the Royal Society B: Biological Sciences 371(1708): 20160001. 10.1098/rstb.2016.0011 PMC5062100 28080969

[bibr13-23982128211007772] Basso L Bönke L Aust S, et al. (2020) Antidepressant and neurocognitive effects of serial ketamine administration versus ECT in depressed patients. Journal of Psychiatric Research 123: 1–8. DOI: 10.1016/j.jpsychires.2020.01.002. 31981856 10.1016/j.jpsychires.2020.01.002

[bibr14-23982128211007772] Bauer SM Schanda H Karakula H, et al. (2011) Culture and the prevalence of hallucinations in schizophrenia. Comprehensive Psychiatry 52(3): 319–325. 21497227 10.1016/j.comppsych.2010.06.008

[bibr15-23982128211007772] Beck AT Rush J Shaw BF, et al. (1979) Cognitive Therapy of Depression. New York, NY: The Guilford Press.

[bibr16-23982128211007772] Béïque JC Imad M Mladenovic L, et al. (2007) Mechanism of the 5-hydroxytryptamine 2A receptor-mediated facilitation of synaptic activity in prefrontal cortex. Proceedings of the National Academy of Sciences of the United States of America 104(23): 9870–9875. 17535909 10.1073/pnas.0700436104 PMC1887564

[bibr17-23982128211007772] Benedetti F Barbini B Campori E, et al. (1996) Dopamine agonist amineptine prevents the antidepressant effect of sleep deprivation. Psychiatry Research 65(3): 179–184. 9029666 10.1016/s0165-1781(96)03000-4

[bibr18-23982128211007772] Benedetti F Campori E Barbini B, et al. (2001) Dopaminergic augmentation of sleep deprivation effects in bipolar depression. Psychiatry Research 104(3): 239–246. 11728613 10.1016/s0165-1781(01)00332-8

[bibr19-23982128211007772] Berman RM Cappiello A Anand A, et al. (2000) Antidepressant effects of ketamine in depressed patients. Biological Psychiatry 47(4): 351–354. 10686270 10.1016/s0006-3223(99)00230-9

[bibr20-23982128211007772] Bobo WV Voort JLV Croarkin PE, et al. (2016) Ketamine for treatment-resistant unipolar and bipolar major depression: Critical review and implications for clinical practice. Depression and Anxiety 33(8): 698–710. 27062450 10.1002/da.22505

[bibr21-23982128211007772] Boland EM Rao H Dinges DF, et al. (2017) Meta-analysis of the antidepressant effects of acute sleep deprivation. The Journal of Clinical Psychiatry 78(8): e1020–e1034. 10.4088/JCP.16r11332 28937707

[bibr22-23982128211007772] Bosch OG Rihm JS Scheidegger M, et al. (2013) Sleep deprivation increases dorsal nexus connectivity to the dorsolateral prefrontal cortex in humans. Proceedings of the National Academy of Sciences 110(48): 19597–19602. 10.1073/pnas.1317010110 PMC3845164 24218598

[bibr23-23982128211007772] Bowers MB Freedman DX (1966) ‘Psychedelic’ experiences in acute psychoses. Archives of General Psychiatry 15(3): 240–248. 5911238 10.1001/archpsyc.1966.01730150016003

[bibr24-23982128211007772] Browning M Behrens TE Jocham G, et al. (2015) Anxious individuals have difficulty learning the causal statistics of aversive environments. Nature Neuroscience 18(4): 590–596. 25730669 10.1038/nn.3961 PMC4644067

[bibr25-23982128211007772] Bruhn JG De Smet PAGM El-Seedi HR, et al. (2002) Mescaline use for 5700 years for personal use. The Lancet 359: 1866. DOI: 10.1016/S0140-6736(02)08701-9. 10.1016/S0140-6736(02)08701-9 12044415

[bibr26-23982128211007772] Bunney BG Bunney WE (2013) Mechanisms of rapid antidepressant effects of sleep deprivation therapy: Clock genes and circadian rhythms. Biological Psychiatry 73(12): 1164–1171. 22906517 10.1016/j.biopsych.2012.07.020

[bibr27-23982128211007772] Carhart-Harris RL Friston KJ (2019) REBUS and the anarchic brain: Toward a unified model of the brain action of psychedelics. Pharmacological Reviews 71(3): 316–344. 31221820 10.1124/pr.118.017160 PMC6588209

[bibr28-23982128211007772] Carhart-Harris RL Bolstridge M Day CMJ, et al. (2018a) Psilocybin with psychological support for treatment-resistant depression: Six-month follow-up. Psychopharmacology 235(2): 399–408. 29119217 10.1007/s00213-017-4771-x PMC5813086

[bibr29-23982128211007772] Carhart-Harris RL Bolstridge M Rucker J, et al. (2016) Psilocybin with psychological support for treatment-resistant depression: An open-label feasibility study. The Lancet Psychiatry 3(7): 619–627. 27210031 10.1016/S2215-0366(16)30065-7

[bibr30-23982128211007772] Carhart-Harris RL Roseman L Bolstridge M, et al. (2017) Psilocybin for treatment-resistant depression: FMRI-measured brain mechanisms. Scientific Reports 7(1): 13187. 29030624 10.1038/s41598-017-13282-7 PMC5640601

[bibr31-23982128211007772] Carhart-Harris RL Roseman L Haijen E, et al. (2018b) Psychedelics and the essential importance of context. Journal of Psychopharmacology 32(7): 725–731. 29446697 10.1177/0269881118754710

[bibr32-23982128211007772] Carter OL Hasler F Pettigrew JD, et al. (2007) Psilocybin links binocular rivalry switch rate to attention and subjective arousal levels in humans. Psychopharmacology 195(3): 415–424. 17874073 10.1007/s00213-007-0930-9

[bibr33-23982128211007772] Carter OL Pettigrew JD Hasler F, et al. (2005) Modulating the rate and rhythmicity of perceptual rivalry alternations with the mixed 5-HT2A and 5-HT1A agonist psilocybin. Neuropsychopharmacology 30(6): 1154–1162. 15688092 10.1038/sj.npp.1300621

[bibr34-23982128211007772] Cassidy CM Balsam PD Weinstein JJ, et al. (2018) A perceptual inference mechanism for hallucinations linked to striatal dopamine. Current Biology 28(4): 503.e4–514.e4. 10.1016/j.cub.2017.12.059 PMC5820222 29398218

[bibr35-23982128211007772] Cedernaes J Brandell J Ros O, et al. (2014) Increased impulsivity in response to food cues after sleep loss in healthy young men. Obesity 22(8): 1786–1791. 24839251 10.1002/oby.20786 PMC4314688

[bibr36-23982128211007772] Chapman J (1966) The early symptoms of schizophrenia. British Journal of Psychiatry 112(484): 225–251. 10.1192/bjp.112.484.225 4957283

[bibr37-23982128211007772] Chekroud AM (2015) Unifying treatments for depression: An application of the Free Energy Principle. Frontiers in Psychology 6: 153. DOI: 10.3389/fpsyg.2015.00153. 25750630 10.3389/fpsyg.2015.00153 PMC4335302

[bibr38-23982128211007772] Clark A (2015) Radical predictive processing. The Southern Journal of Philosophy 53(S1): 3–27.

[bibr39-23982128211007772] Clark JE Watson S Friston KJ (2018) What is mood? A computational perspective. Psychological Medicine 48(14): 2277–2284. 29478431 10.1017/S0033291718000430 PMC6340107

[bibr40-23982128211007772] Coifman KG Summers CB (2019) Understanding emotion inflexibility in risk for affective disease: Integrating current research and finding a path forward. Frontiers in Psychology 10: 392. DOI: 10.3389/fpsyg.2019.00392. 30873087 10.3389/fpsyg.2019.00392 PMC6402431

[bibr41-23982128211007772] Cole DM Diaconescu AO Pfeiffer UJ, et al. (2020) Atypical processing of uncertainty in individuals at risk for psychosis. NeuroImage: Clinical 26(239): 102239. 32182575 10.1016/j.nicl.2020.102239 PMC7076146

[bibr42-23982128211007772] Corlett PR Honey GD Aitken MRF, et al. (2006) Frontal responses during learning predict vulnerability to the psychotogenic effects of ketamine: Linking cognition, brain activity, and psychosis. Archives of General Psychiatry 63(6): 611–621. 16754834 10.1001/archpsyc.63.6.611

[bibr43-23982128211007772] Corlett PR Honey GD Fletcher PC (2007) From prediction error to psychosis: Ketamine as a pharmacological model of delusions. Journal of Psychopharmacology 21(3): 238–252. 17591652 10.1177/0269881107077716

[bibr44-23982128211007772] Corlett PR Honey GD Fletcher PC (2016) Prediction error, ketamine and psychosis: An updated model. Journal of Psychopharmacology 30(11): 1145–1155. 27226342 10.1177/0269881116650087 PMC5105325

[bibr45-23982128211007772] Davis RN Nolen-Hoeksema S (2000) Cognitive inflexibility among ruminators and nonruminators. Cognitive Therapy and Research 24(6): 699–711.

[bibr46-23982128211007772] de Osório FL Sanches RF Macedo LR, et al. (2015) Antidepressant effects of a single dose of ayahuasca in patients with recurrent depression: A preliminary report. Revista Brasileira de Psiquiatria 37(1): 13–20. 25806551 10.1590/1516-4446-2014-1496

[bibr47-23982128211007772] Demos KE Hart CN Sweet LH, et al. (2016) Partial sleep deprivation impacts impulsive action but not impulsive decision-making. Physiology and Behavior 164(Pt A): 214–219. 27267950 10.1016/j.physbeh.2016.06.003 PMC5612429

[bibr48-23982128211007772] Deserno L Boehme R Mathys C, et al. (2020) Volatility estimates increase choice switching and relate to prefrontal activity in schizophrenia. Biological Psychiatry: Cognitive Neuroscience and Neuroimaging 5(2): 173–183. 31937449 10.1016/j.bpsc.2019.10.007

[bibr49-23982128211007772] Diamond A (2013) Executive functions. Annual Review of Psychology 64: 135–168. DOI: 10.1146/annurev-psych-113011-143750. 10.1146/annurev-psych-113011-143750 PMC4084861 23020641

[bibr50-23982128211007772] Diederen KMJ Ziauddeen H Vestergaard MD, et al. (2017) Dopamine modulates adaptive prediction error coding in the human midbrain and striatum. Journal of Neuroscience 37(7): 1708–1720. 28202786 10.1523/JNEUROSCI.1979-16.2016 PMC5320604

[bibr51-23982128211007772] Dima D Dietrich DE Dillo W, et al. (2010) Impaired top-down processes in schizophrenia: A DCM study of ERPs. NeuroImage 52(3): 824–832. 20056155 10.1016/j.neuroimage.2009.12.086

[bibr52-23982128211007772] Dore J Turnipseed B Dwyer S, et al. (2019) Ketamine assisted psychotherapy (KAP): Patient demographics, clinical data and outcomes in three large practices administering ketamine with psychotherapy. Journal of Psychoactive Drugs 51(2): 189–198. 30917760 10.1080/02791072.2019.1587556

[bibr53-23982128211007772] Ebert D Feistel H Kaschka W, et al. (1994) Single photon emission computerized tomography assessment of cerebral dopamine D2 receptor blockade in depression before and after sleep deprivation-preliminary results. Biological Psychiatry 35(11): 880–885. 8054411 10.1016/0006-3223(94)90024-8

[bibr54-23982128211007772] Edwards S Koob GF (2012) Experimental psychiatric illness and drug abuse models: From human to animal, an overview. Methods in Molecular Biology 829: 31–48. DOI: 10.1007/978-1-61779-458-2_2. 22231805 10.1007/978-1-61779-458-2_2 PMC3285446

[bibr55-23982128211007772] Feinstein JS Khalsa SS Yeh H, et al. (2018) Examining the short-term anxiolytic and antidepressant effect of Floatation-REST. PLoS ONE 13(2): e0190292. 10.1371/journal.pone.0190292 PMC5796691 29394251

[bibr56-23982128211007772] Fletcher PC Frith CD (2009) Perceiving is believing: A Bayesian approach to explaining the positive symptoms of schizophrenia. Nature Reviews Neuroscience 10(1): 48–58. 19050712 10.1038/nrn2536

[bibr57-23982128211007772] Friston K (2009) The free-energy principle: A rough guide to the brain? Trends in Cognitive Sciences 13(7): 293–301. 19559644 10.1016/j.tics.2009.04.005

[bibr58-23982128211007772] Friston K (2010) The free-energy principle: A unified brain theory? Nature Reviews Neuroscience 11(2): 127–138. 20068583 10.1038/nrn2787

[bibr59-23982128211007772] Friston K Kiebel S (2009) Predictive coding under the free-energy principle. Philosophical Transactions of the Royal Society B: Biological Sciences 364(1521): 1211–1221. 10.1098/rstb.2008.0300 PMC2666703 19528002

[bibr60-23982128211007772] Gao M Rejaei D Liu H (2016) Ketamine use in current clinical practice. Acta Pharmacologica Sinica 37(7): 865–872. 27018176 10.1038/aps.2016.5 PMC4933765

[bibr61-23982128211007772] González-Maeso J Weisstaub NV Zhou M, et al. (2007) Hallucinogens recruit specific cortical 5-HT2A receptor-mediated signaling pathways to affect behavior. Neuron 53(3): 439–452. 17270739 10.1016/j.neuron.2007.01.008

[bibr62-23982128211007772] Gouzoulis-Mayfrank E Habermeyer E Hermle L, et al. (1998) Hallucinogenic drug induced states resemble acute endogenous psychoses: Results of an empirical study. European Psychiatry 13(8): 399–406. 19698655 10.1016/S0924-9338(99)80686-5

[bibr63-23982128211007772] Gujar N Yoo S-S Hu P, et al. (2011) Sleep deprivation amplifies reactivity of brain reward networks, biasing the appraisal of positive emotional experiences. Journal of Neuroscience 31(12): 4466–4474. 21430147 10.1523/JNEUROSCI.3220-10.2011 PMC3086142

[bibr64-23982128211007772] Haarsma J Fletcher PC Griffin JD, et al. (2020a) Precision weighting of cortical unsigned prediction error signals benefits learning, is mediated by dopamine, and is impaired in psychosis. Molecular Psychiatry. Epub ahead of print 24 June. DOI: 10.1038/s41380-020-0803-8. PMC8589669 32576965

[bibr65-23982128211007772] Haarsma J Knolle F Griffin JD, et al. (2020b) Influence of prior beliefs on perception in early psychosis: Effects of illness stage and hierarchical level of belief. Journal of Abnormal Psychology 129(6): 581–598. 32757602 10.1037/abn0000494 PMC7409392

[bibr66-23982128211007772] Haarsma J Kok P Browning M (2020c) The promise of layer-specific neuroimaging for testing predictive coding theories of psychosis. Schizophrenia Research. Epub ahead of print 13 November. DOI: 10.31234/osf.io/xeyv7. PMC9241988 33199171

[bibr67-23982128211007772] Hales CA Houghton CJ Robinson ESJ (2017) Behavioural and computational methods reveal differential effects for how delayed and rapid onset antidepressants effect decision making in rats. European Neuropsychopharmacology 27(12): 1268–1280. 29100819 10.1016/j.euroneuro.2017.09.008 PMC5720479

[bibr68-23982128211007772] Harmer CJ Goodwin GM Cowen PJ (2009) Why do antidepressants take so long to work? A cognitive neuropsychological model of antidepressant drug action. British Journal of Psychiatry 195(2): 102–108. 10.1192/bjp.bp.108.051193 19648538

[bibr69-23982128211007772] Heuschkel K Kuypers KPC (2020) Depression, mindfulness, and psilocybin: Possible complementary effects of mindfulness meditation and psilocybin in the treatment of depression. A review. Frontiers in Psychiatry 11: 224. DOI: 10.3389/fpsyt.2020.00224. 32296353 10.3389/fpsyt.2020.00224 PMC7136554

[bibr70-23982128211007772] Hoch PH Cattell JP Pennes HH (1952) Effect of drugs; theoretical considerations from a psychological viewpoint. The American Journal of Psychiatry 108(8): 585–589. 14903184 10.1176/ajp.108.8.585

[bibr71-23982128211007772] Hohwy J (2013) The Predictive Mind. Oxford: Oxford University Press.

[bibr72-23982128211007772] Houston RJ Bauer LO Hesselbrock VM (2004) P300 evidence of cognitive inflexibility in female adolescents at risk for recurrent depression. Progress in Neuro-Psychopharmacology and Biological Psychiatry 28(3): 529–536. 15093961 10.1016/j.pnpbp.2004.01.004

[bibr73-23982128211007772] Iglesias S Mathys C Brodersen KH, et al. (2013) Hierarchical prediction errors in midbrain and basal forebrain during sensory learning. Neuron 80(2): 519–530. 24139048 10.1016/j.neuron.2013.09.009

[bibr74-23982128211007772] Irwin MR Olmstead R Carroll JE (2016) Sleep disturbance, sleep duration, and inflammation: A systematic review and meta-analysis of cohort studies and experimental sleep deprivation. Biological Psychiatry 80(1): 40–52. 26140821 10.1016/j.biopsych.2015.05.014 PMC4666828

[bibr75-23982128211007772] Joffily M Coricelli G (2013) Emotional valence and the free-energy principle. PLoS Computational Biology 9(6): e1003094. 10.1371/journal.pcbi.1003094 PMC3681730 23785269

[bibr76-23982128211007772] Joormann J Levens SM Gotlib IH (2011) Sticky thoughts: Depression and rumination are associated with difficulties manipulating emotional material in working memory. Psychological Science 22(8): 979–983. 21742932 10.1177/0956797611415539 PMC11862919

[bibr77-23982128211007772] Kaelen M Roseman L Kahan J, et al. (2016) LSD modulates music-induced imagery via changes in parahippocampal connectivity. European Neuropsychopharmacology 26(7): 1099–1109. 27084302 10.1016/j.euroneuro.2016.03.018

[bibr78-23982128211007772] Kaplan CM Saha D Molina JL, et al. (2016) Estimating changing contexts in schizophrenia. Brain 139(7): 2082–2095. 27217338 10.1093/brain/aww095 PMC4939701

[bibr79-23982128211007772] Kanen JW Luo Q Kandroodi MR, et al. (2021) Effect of lysergic acid diethylamide (LSD) on reinforcement learning in humans. Pre-print. bioRxiv: 2020.12.04.412189. DOI: 10.1101/2020.12.04.412189. 10.1017/S0033291722002963 PMC10600934 36411719

[bibr80-23982128211007772] Killgore WDS (2010) Effects of sleep deprivation on cognition. Progress in Brain Research 185: 105–129. DOI: 10.3389/fpsyt.2020.00224. 21075236 10.1016/B978-0-444-53702-7.00007-5

[bibr81-23982128211007772] Killgore WDS Balkin TJ Wesensten NJ (2006) Impaired decision making following 49 h of sleep deprivation. Journal of Sleep Research 15(1): 7–13. 16489997 10.1111/j.1365-2869.2006.00487.x

[bibr82-23982128211007772] Kishimoto T Chawla JM Hagi K, et al. (2016) Single-dose infusion ketamine and non-ketamine N-methyl-d-aspartate receptor antagonists for unipolar and bipolar depression: A meta-analysis of efficacy, safety and time trajectories. Psychological Medicine 46(7): 1459–1472. 26867988 10.1017/S0033291716000064 PMC5116384

[bibr83-23982128211007772] Kjellgren A Lyden F Norlander T (2008) Sensory isolation in flotation tanks: Altered states of consciousness and effects on well-being. The Qualitative Report 13(4): 636–656.

[bibr84-23982128211007772] Kok P Bains LJ Van Mourik T, et al. (2016) Selective activation of the deep layers of the human primary visual cortex by top-down feedback. Current Biology 26(3): 371–376. 26832438 10.1016/j.cub.2015.12.038

[bibr85-23982128211007772] Kometer M Cahn BR Andel D, et al. (2011) The 5-HT2A/1A agonist psilocybin disrupts modal object completion associated with visual hallucinations. Biological Psychiatry 69(5): 399–406. 21126732 10.1016/j.biopsych.2010.10.002

[bibr86-23982128211007772] Krause AJ Simon E Ben Mander BA, et al. (2017) The sleep-deprived human brain. Nature Reviews Neuroscience 18(7): 404–418. 28515433 10.1038/nrn.2017.55 PMC6143346

[bibr87-23982128211007772] Krystal JH Abi-Saab W Perry E, et al. (2005) Preliminary evidence of attenuation of the disruptive effects of the NMDA glutamate receptor antagonist, ketamine, on working memory by pretreatment with the group II metabotropic glutamate receptor agonist, LY354740, in healthy human subjects. Psychopharmacology 179(1): 303–309. 15309376 10.1007/s00213-004-1982-8

[bibr88-23982128211007772] Krystal JH Karper LP Seibyl JP, et al. (1994) Subanesthetic effects of the noncompetitive NMDA antagonist, ketamine, in humans: Psychotomimetic, perceptual, cognitive, and neuroendocrine responses. Archives of General Psychiatry 51(3): 199–214. 8122957 10.1001/archpsyc.1994.03950030035004

[bibr89-23982128211007772] Krystal JH Sanacora G Duman RS (2013) Rapid-acting glutamatergic antidepressants: The path to ketamine and beyond. Biological Psychiatry 73(12): 1133–1141. 23726151 10.1016/j.biopsych.2013.03.026 PMC3671489

[bibr90-23982128211007772] Kube T Rozenkrantz L (2020) When beliefs face reality: An integrative review of belief updating in mental health and illness. Perspectives on Psychological Science 16(2): 247–274. 32818386 10.1177/1745691620931496

[bibr91-23982128211007772] Kuijpers HJH Van Der Heijden FMMA Tuinier S, et al. (2007) Meditation-induced psychosis. Psychopathology 40(6): 461–464. 17848828 10.1159/000108125

[bibr92-23982128211007772] Lally N Nugent AC Luckenbaugh DA, et al. (2014) Anti-anhedonic effect of ketamine and its neural correlates in treatment-resistant bipolar depression. Translational Psychiatry 4: e469. DOI: 10.1038/tp.2014.105. 10.1038/tp.2014.105 PMC4350513 25313512

[bibr93-23982128211007772] Lally N Nugent AC Luckenbaugh DA, et al. (2015) Neural correlates of change in major depressive disorder anhedonia following open-label ketamine. Journal of Psychopharmacology 29(5): 596–607. 25691504 10.1177/0269881114568041 PMC5116382

[bibr94-23982128211007772] Leahy RL Tirch DD Melwani PS (2012) Processes underlying depression: Risk aversion, emotional schemas, and psychological flexibility. International Journal of Cognitive Therapy 5(4): 362–379.

[bibr95-23982128211007772] Leptourgos P Fortier-Davy M Carhart-Harris R, et al. (2020) Hallucinations under psychedelics and in the schizophrenia spectrum: An interdisciplinary and multiscale comparison. Schizophrenia Bulletin 46(6): 1396–1408. 32944778 10.1093/schbul/sbaa117 PMC7707069

[bibr96-23982128211007772] Leuner H Holfeld H (1962) Ergebnisse und probleme der psychotherapie mit hilfe von LSD-25 und verwandten substanzen. European Neurology 143(6): 379–391.

[bibr97-23982128211007772] Lewis KS Gordon-Smith K Forty L, et al. (2017) Sleep loss as a trigger of mood episodes in bipolar disorder: Individual differences based on diagnostic subtype and gender. British Journal of Psychiatry 211(3): 169–174. 10.1192/bjp.bp.117.202259 PMC5579327 28684405

[bibr98-23982128211007772] Li N Lee B Liu RJ, et al. (2010) mTOR-dependent synapse formation underlies the rapid antidepressant effects of NMDA antagonists. Science 329(5994): 959–964. 20724638 10.1126/science.1190287 PMC3116441

[bibr99-23982128211007772] Loix S De Kock M Henin P (2011) The anti-inflammatory effects of ketamine: State of the art. Acta Anaesthesiologica Belgica 62(1): 47–58. 21612145

[bibr100-23982128211007772] Lopez-Rodriguez F Wilson CL Maidment NT, et al. (2003) Total sleep deprivation increases extracellular serotonin in the rat hippocampus. Neuroscience 121(2): 523–530. 14522011 10.1016/s0306-4522(03)00335-x

[bibr101-23982128211007772] Loureiro JRA Leaver A Vasavada M, et al. (2020) Modulation of amygdala reactivity following rapidly acting interventions for major depression. Human Brain Mapping 41(7): 1699–1710. 32115848 10.1002/hbm.24895 PMC7268016

[bibr102-23982128211007772] Lowe CJ Safati A Hall PA (2017) The neurocognitive consequences of sleep restriction: A meta-analytic review. Neuroscience and Biobehavioral Reviews 80: 586–604. DOI: 10.1016/j.neubiorev.2017.07.010. 28757454 10.1016/j.neubiorev.2017.07.010

[bibr103-23982128211007772] McDermott CM Hardy MN Bazan NG, et al. (2006) Sleep deprivation-induced alterations in excitatory synaptic transmission in the CA1 region of the rat hippocampus. Journal of Physiology 570(3): 553–565. 10.1113/jphysiol.2005.093781 PMC1479879 16322058

[bibr104-23982128211007772] McGirr A Berlim MT Bond DJ, et al. (2015) A systematic review and meta-analysis of randomized, double-blind, placebo-controlled trials of ketamine in the rapid treatment of major depressive episodes. Psychological Medicine 45(4): 693–704. 25010396 10.1017/S0033291714001603

[bibr105-23982128211007772] Malhotra AK Pinals DA Weingartner H, et al. (1996) NMDA receptor function and human cognition: The effects of ketamine in healthy volunteers. Neuropsychopharmacology 14(5): 301–307. 8703299 10.1016/0893-133X(95)00137-3

[bibr106-23982128211007772] Mathys CD Daunizeau J Friston KJ, et al. (2011) A Bayesian foundation for individual learning under uncertainty. Frontiers in Human Neuroscience 5: 39. DOI: 10.3389/fnhum.2011.00039. 21629826 10.3389/fnhum.2011.00039 PMC3096853

[bibr107-23982128211007772] Mathys CD Lomakina EI Daunizeau J, et al. (2014) Uncertainty in perception and the Hierarchical Gaussian filter. Frontiers in Human Neuroscience 8: 825. DOI: 10.3389/fnhum.2014.00825. 25477800 10.3389/fnhum.2014.00825 PMC4237059

[bibr108-23982128211007772] Mion G (2017) History of anaesthesia: The ketamine story – Past, present and future. European Journal of Anaesthesiology 34(9): 571–575. 28731926 10.1097/EJA.0000000000000638

[bibr109-23982128211007772] Moghaddam B Adams B Verma A, et al. (1997) Activation of glutamatergic neurotransmission by ketamine: A novel step in the pathway from NMDA receptor blockade to dopaminergic and cognitive disruptions associated with the prefrontal cortex. Journal of Neuroscience 17(8): 2921–2927. 9092613 10.1523/JNEUROSCI.17-08-02921.1997 PMC6573099

[bibr110-23982128211007772] Monteggia LM Gideons E Kavalali ET (2013) The role of eukaryotic elongation factor 2 kinase in rapid antidepressant action of ketamine. Biological Psychiatry 73(12): 1199–1203. 23062356 10.1016/j.biopsych.2012.09.006 PMC3574622

[bibr111-23982128211007772] Moore JW Turner DC Corlett PR, et al. (2011) Ketamine administration in healthy volunteers reproduces aberrant agency experiences associated with schizophrenia. Cognitive Neuropsychiatry 16(4): 364–381. 21302161 10.1080/13546805.2010.546074 PMC3144485

[bibr112-23982128211007772] Moran RJ Campo P Symmonds M, et al. (2013) Free energy, precision and learning: The role of cholinergic neuromodulation. Journal of Neuroscience 33(19): 8227–8236. 23658161 10.1523/JNEUROSCI.4255-12.2013 PMC4235126

[bibr113-23982128211007772] Morris LS Costi S Tan A, et al. (2020) Ketamine normalizes subgenual cingulate cortex hyper-activity in depression. Neuropsychopharmacology 45(6): 975–981. 31896116 10.1038/s41386-019-0591-5 PMC7162851

[bibr114-23982128211007772] Mueller F Lenz C Dolder PC, et al. (2017) Acute effects of LSD on amygdala activity during processing of fearful stimuli in healthy subjects. Translational Psychiatry 7(4): e1084–e1085. 28375205 10.1038/tp.2017.54 PMC5416695

[bibr115-23982128211007772] Murck H Schubert MI Schmid D, et al. (2009) The glutamatergic system and its relation to the clinical effect of therapeutic-sleep deprivation in depression – An MR spectroscopy study. Journal of Psychiatric Research 43(3): 175–180. 18533184 10.1016/j.jpsychires.2008.04.009

[bibr116-23982128211007772] Muttoni S Ardissino M John C (2019) Classical psychedelics for the treatment of depression and anxiety: A systematic review. Journal of Affective Disorders 258: 11–24. DOI: 10.1016/j.jad.2019.07.076. 31382100 10.1016/j.jad.2019.07.076

[bibr117-23982128211007772] Newport DJ Carpenter LL McDonald WM, et al. (2015) Ketamine and other NMDA antagonists: Early clinical trials and possible mechanisms in depression. American Journal of Psychiatry 172(10): 950–966. 10.1176/appi.ajp.2015.15040465 26423481

[bibr118-23982128211007772] Nugent AC Ballard ED Gould TD, et al. (2019) Ketamine has distinct electrophysiological and behavioral effects in depressed and healthy subjects. Molecular Psychiatry 24(7): 1040–1052. 29487402 10.1038/s41380-018-0028-2 PMC6111001

[bibr119-23982128211007772] Olson EA Weber M Rauch SL, et al. (2016) Daytime sleepiness is associated with reduced integration of temporally distant outcomes on the Iowa gambling task. Behavioral Sleep Medicine 14(2): 200–211. 25396689 10.1080/15402002.2014.974182

[bibr120-23982128211007772] Pak A Ryu E Li C, et al. (2020) Top-down feedback controls the cortical representation of illusory contours in mouse primary visual cortex. The Journal of Neuroscience: The Official Journal of the Society for Neuroscience 40(3): 648–660. 31792152 10.1523/JNEUROSCI.1998-19.2019 PMC6961994

[bibr121-23982128211007772] Park C Pan Z Brietzke E, et al. (2018) Predicting antidepressant response using early changes in cognition: A systematic review. Behavioural Brain Research 353: 154–160. DOI: 10.1016/j.bbr.2018.07.011. 30031025 10.1016/j.bbr.2018.07.011

[bibr122-23982128211007772] Pflug B Tölle R (1971) Therapy of endogenous depressions using sleep deprivation. Practical and theoretical consequences. Der Nervenarzt 42(3): 117–124. 5550102

[bibr123-23982128211007772] Piray P Daw ND (2020) A simple model for learning in volatile environments. PLoS Computational Biology 16(7): e1007963. 10.1371/journal.pcbi.1007963 PMC7329063 32609755

[bibr124-23982128211007772] Pomarol-Clotet E Honey GD Murray GK, et al. (2006) Psychological effects of ketamine in healthy volunteers: Phenomenological study. British Journal of Psychiatry 189: 173–179. DOI: 10.1192/bjp.bp.105.015263. 10.1192/bjp.bp.105.015263 PMC3838932 16880489

[bibr125-23982128211007772] Puig MV Celada P Díaz-Mataix L, et al. (2003) In vivo modulation of the activity of pyramidal neurons in the rat medial prefrontal cortex by 5-HT2A receptors: Relationship to thalamocortical afferents. Cerebral Cortex 13(8): 870–882. 12853374 10.1093/cercor/13.8.870

[bibr126-23982128211007772] Pulcu E Browning M (2017) Affective bias as a rational response to the statistics of rewards and punishments. ELife 6: e27879. 10.7554/eLife.27879 PMC5633345 28976304

[bibr127-23982128211007772] Pulcu E Browning M (2019) The misestimation of uncertainty in affective disorders. Trends in Cognitive Sciences 23(10): 865–875. DOI: 10.7554/eLife.27879. 31431340 10.1016/j.tics.2019.07.007

[bibr128-23982128211007772] Quednow BB Kometer M Geyer MA, et al. (2012) Psilocybin-induced deficits in automatic and controlled inhibition are attenuated by Ketanserin in healthy human volunteers. Neuropsychopharmacology 37(3): 630–640. 21956447 10.1038/npp.2011.228 PMC3260978

[bibr129-23982128211007772] Rao RPN Ballard DH (1999) Predictive coding in the visual cortex: A functional interpretation of some extra-classical receptive-field effects. Nature Neuroscience 2(1): 79–87. 10195184 10.1038/4580

[bibr130-23982128211007772] Reiff CM Richman EE Nemeroff CB, et al. (2020) Psychedelics and psychedelic-assisted psychotherapy. American Journal of Psychiatry 177(5): 391–410. 10.1176/appi.ajp.2019.19010035 32098487

[bibr131-23982128211007772] Remijnse PL Nielen MMA Van Balkom AJLM, et al. (2009) Differential frontal-striatal and paralimbic activity during reversal learning in major depressive disorder and obsessive-compulsive disorder. Psychological Medicine 39(9): 1503–1518. 19171077 10.1017/S0033291708005072

[bibr132-23982128211007772] Remijnse PL van den Heuvel OA Nielen MMA, et al. (2013) Cognitive inflexibility in obsessive-compulsive disorder and major depression is associated with distinct neural correlates. PLoS ONE 8(4): e59600. 10.1371/journal.pone.0059600 PMC3634812 23637737

[bibr133-23982128211007772] Riemann D Krone LB Wulff K, et al. (2020) Sleep, insomnia, and depression. Neuropsychopharmacology 45(1): 74–89. 31071719 10.1038/s41386-019-0411-y PMC6879516

[bibr134-23982128211007772] Rock PL Roiser JP Riedel WJ, et al. (2014) Cognitive impairment in depression: A systematic review and meta-analysis. Psychological Medicine 44(10): 2029–2040. 24168753 10.1017/S0033291713002535

[bibr135-23982128211007772] Roseman L Demetriou L Wall MB, et al. (2018) Increased amygdala responses to emotional faces after psilocybin for treatment-resistant depression. Neuropharmacology 142: 263–269. DOI: 10.1016/j.neuropharm.2017.12.041. 29288686 10.1016/j.neuropharm.2017.12.041

[bibr136-23982128211007772] Salomon RM Delgado PL Licinio J, et al. (1994) Effects of sleep deprivation on serotonin function in depression. Biological Psychiatry 36(12): 840–846. 7893848 10.1016/0006-3223(94)90595-9

[bibr137-23982128211007772] Scheidegger M Henning A Walter M, et al. (2016) Ketamine administration reduces Amygdalo-hippocampal reactivity to emotional stimulation. Human Brain Mapping 37(5): 1941–1952. 26915535 10.1002/hbm.23148 PMC6867525

[bibr138-23982128211007772] Scheidegger M Walter M Lehmann M, et al. (2012) Ketamine decreases resting state functional network connectivity in healthy subjects: Implications for antidepressant drug action. PLoS ONE 7(9): e44799. 10.1371/journal.pone.0044799 PMC3461985 23049758

[bibr139-23982128211007772] Schmack K de Castro AGC Rothkirch M, et al. (2013) Delusions and the role of beliefs in perceptual inference. Journal of Neuroscience 33(34): 13701–13712. 23966692 10.1523/JNEUROSCI.1778-13.2013 PMC6618656

[bibr140-23982128211007772] Schmid Y Enzler F Gasser P, et al. (2015) Acute effects of lysergic acid diethylamide in healthy subjects. Biological Psychiatry 78(8): 544–553. 25575620 10.1016/j.biopsych.2014.11.015

[bibr141-23982128211007772] Schulte W (1966) Kombinierte Psycho- und Pharmakotherapie bei Melancholikern. In: Petrilowitsch N Kranz H (eds) Probleme der Pharmakopsychiatrischen Kombinations- und Langzeitbehandlung. Basel: S. Karger AG, pp. 150–169.

[bibr142-23982128211007772] Solé B Jiménez E Martinez-Aran A, et al. (2015) Cognition as a target in major depression: New developments. European Neuropsychopharmacology 25(2): 231–247. 25640673 10.1016/j.euroneuro.2014.12.004

[bibr143-23982128211007772] Sos P Klirova M Novak T, et al. (2013) Relationship of ketamine’s antidepressant and psychotomimetic effects in unipolar depression. Neuro Endocrinology Letters 34(4): 287–293. 23803871

[bibr144-23982128211007772] Spratling MW (2008) Predictive coding as a model of biased competition in visual attention. Vision Research 48(12): 1391–1408. 18442841 10.1016/j.visres.2008.03.009

[bibr145-23982128211007772] Spratling MW (2010) Predictive coding as a model of response properties in cortical area V1. Journal of Neuroscience 30(9): 3531–3543. 20203213 10.1523/JNEUROSCI.4911-09.2010 PMC6634102

[bibr146-23982128211007772] Spratling MW (2017) A review of predictive coding algorithms. Brain and Cognition 112: 92–97. DOI: 10.1016/j.bandc.2015.11.003. 26809759 10.1016/j.bandc.2015.11.003

[bibr147-23982128211007772] Steinberg H Hegerl U (2014) Johann Christian August Heinroth on sleep deprivation as a therapeutic option for depressive disorders. Sleep Medicine 15(9): 1159–1164. 24994565 10.1016/j.sleep.2014.03.027

[bibr148-23982128211007772] Stephan KE Baldeweg T Friston KJ (2006) Synaptic plasticity and dysconnection in schizophrenia. Biological Psychiatry 59(10): 929–939. 16427028 10.1016/j.biopsych.2005.10.005

[bibr149-23982128211007772] Sterpenich V Vidal S Hofmeister J, et al. (2019) Increased reactivity of the mesolimbic reward system after ketamine injection in patients with treatment-resistant major depressive disorder. Anesthesiology 130(6): 923–935. 31021848 10.1097/ALN.0000000000002667

[bibr150-23982128211007772] Sterzer P Adams RA Fletcher P, et al. (2018) The predictive coding account of psychosis. Biological Psychiatry 84(9): 634–643. 30007575 10.1016/j.biopsych.2018.05.015 PMC6169400

[bibr151-23982128211007772] Stuart SA Butler P Munafò MR, et al. (2015) Distinct neuropsychological mechanisms may explain delayed-versus rapid-onset antidepressant efficacy. Neuropsychopharmacology 40(9): 2165–2174. 25740288 10.1038/npp.2015.59 PMC4487826

[bibr152-23982128211007772] Teufel C Subramaniam N Dobler V, et al. (2015) Shift toward prior knowledge confers a perceptual advantage in early psychosis and psychosis-prone healthy individuals. Proceedings of the National Academy of Sciences of the United States of America 112(43): 13401–13406. 26460044 10.1073/pnas.1503916112 PMC4629373

[bibr153-23982128211007772] Timmermann C Spriggs MJ Kaelen M, et al. (2018) LSD modulates effective connectivity and neural adaptation mechanisms in an auditory oddball paradigm. Neuropharmacology 142: 251–262. DOI: 10.1016/j.neuropharm.2017.10.039. 29101022 10.1016/j.neuropharm.2017.10.039

[bibr154-23982128211007772] Valton V Karvelis P Richards KL, et al. (2019) Acquisition of visual priors and induced hallucinations in chronic schizophrenia. Brain 142(8): 2523–2537. 31257444 10.1093/brain/awz171 PMC6734996

[bibr155-23982128211007772] Vinckier F Gaillard R Palminteri S, et al. (2016) Confidence and psychosis: A neuro-computational account of contingency learning disruption by NMDA blockade. Molecular Psychiatry 21(7): 946–955. 26055423 10.1038/mp.2015.73 PMC5414075

[bibr156-23982128211007772] Vollenweider FX Kometer M (2010) The neurobiology of psychedelic drugs: Implications for the treatment of mood disorders. Nature Reviews Neuroscience 11(9): 642–651. 20717121 10.1038/nrn2884

[bibr157-23982128211007772] Vollenweider FX Vontobel P Hell D, et al. (1999) 5–HT modulation of dopamine release in basal ganglia in psilocybin–induced psychosis in man – A PET study with [11C]raclopride. Neuropsychopharmacology 20(5): 424–433. 10192823 10.1016/S0893-133X(98)00108-0

[bibr158-23982128211007772] Waters F Chiu V Atkinson A, et al. (2018) Severe sleep deprivation causes hallucinations and a gradual progression toward psychosis with increasing time awake. Frontiers in Psychiatry 9: 303. DOI: 10.3389/fpsyt.2018.00303. 30042701 10.3389/fpsyt.2018.00303 PMC6048360

[bibr159-23982128211007772] Weber LA Diaconescu AO Mathys C, et al. (2020) Ketamine affects prediction errors about statistical regularities: A computational single-trial analysis of the mismatch negativity. The Journal of Neuroscience: The Official Journal of the Society for Neuroscience 40(29): 5658–5668. 32561673 10.1523/JNEUROSCI.3069-19.2020 PMC7363468

[bibr160-23982128211007772] Wehr TA Sack DA Rosenthal NE (1987) Sleep reduction as a final common pathway in the genesis of mania. American Journal of Psychiatry 144(2): 201–204. 10.1176/ajp.144.2.201 3812788

[bibr161-23982128211007772] Wolf E Kuhn M Norman C, et al. (2015) Synaptic plasticity model of therapeutic sleep deprivation in major depression. Sleep Medicine Reviews 30: 53–62. DOI: 10.1016/j.smrv.2015.11.003. 26803484 10.1016/j.smrv.2015.11.003

[bibr162-23982128211007772] World Health Organization (WHO) (2017) Depression and Other Common Mental Disorders. Geneva: WHO.

[bibr163-23982128211007772] Xu Y Hackett M Carter G, et al. (2016) Effects of low-dose and very low-dose ketamine among patients with major depression: A systematic review and meta-analysis. International Journal of Neuropsychopharmacology 19(4): pyv124. 10.1093/ijnp/pyv124 PMC4851268 26578082

[bibr164-23982128211007772] Zheng W Zhou YL Liu WJ, et al. (2019) Neurocognitive performance and repeated-dose intravenous ketamine in major depressive disorder. Journal of Affective Disorders 246: 241–247. DOI: 10.1016/j.jad.2018.12.005. 30590286 10.1016/j.jad.2018.12.005

